# Uncovering genetic associations in the human diseasome using an endophenotype-augmented disease network

**DOI:** 10.1093/bioinformatics/btae126

**Published:** 2024-03-12

**Authors:** Jakob Woerner, Vivek Sriram, Yonghyun Nam, Anurag Verma, Dokyoon Kim

**Affiliations:** Department of Biostatistics, Epidemiology and Informatics, Perelman School of Medicine, University of Pennsylvania, Philadelphia, PA 19104, United States; Genomics and Computational Biology Graduate Group, Perelman School of Medicine, University of Pennsylvania, Philadelphia, PA 19104, United States; Department of Biostatistics, Epidemiology and Informatics, Perelman School of Medicine, University of Pennsylvania, Philadelphia, PA 19104, United States; Genomics and Computational Biology Graduate Group, Perelman School of Medicine, University of Pennsylvania, Philadelphia, PA 19104, United States; Department of Biostatistics, Epidemiology and Informatics, Perelman School of Medicine, University of Pennsylvania, Philadelphia, PA 19104, United States; Department of Medicine, Perelman School of Medicine, University of Pennsylvania, Philadelphia, PA 19104, United States; Institute for Biomedical Informatics, University of Pennsylvania, Philadelphia, PA 19104, United States; Department of Biostatistics, Epidemiology and Informatics, Perelman School of Medicine, University of Pennsylvania, Philadelphia, PA 19104, United States; Institute for Biomedical Informatics, University of Pennsylvania, Philadelphia, PA 19104, United States

## Abstract

**Motivation:**

Many diseases, particularly cardiometabolic disorders, exhibit complex multimorbidities with one another. An intuitive way to model the connections between phenotypes is with a disease-disease network (DDN), where nodes represent diseases and edges represent associations, such as shared single-nucleotide polymorphisms (SNPs), between pairs of diseases. To gain further genetic understanding of molecular contributors to disease associations, we propose a novel version of the shared-SNP DDN (ssDDN), denoted as ssDDN+, which includes connections between diseases derived from genetic correlations with intermediate endophenotypes. We hypothesize that a ssDDN+ can provide complementary information to the disease connections in a ssDDN, yielding insight into the role of clinical laboratory measurements in disease interactions.

**Results:**

Using PheWAS summary statistics from the UK Biobank, we constructed a ssDDN+ revealing hundreds of genetic correlations between diseases and quantitative traits. Our augmented network uncovers genetic associations across different disease categories, connects relevant cardiometabolic diseases, and highlights specific biomarkers that are associated with cross-phenotype associations. Out of the 31 clinical measurements under consideration, HDL-C connects the greatest number of diseases and is strongly associated with both type 2 diabetes and heart failure. Triglycerides, another blood lipid with known genetic causes in non-mendelian diseases, also adds a substantial number of edges to the ssDDN. This work demonstrates how association with clinical biomarkers can better explain the shared genetics between cardiometabolic disorders. Our study can facilitate future network-based investigations of cross-phenotype associations involving pleiotropy and genetic heterogeneity, potentially uncovering sources of missing heritability in multimorbidities.

**Availability and implementation:**

The generated ssDDN+ can be explored at https://hdpm.biomedinfolab.com/ddn/biomarkerDDN.

## 1 Introduction

Complex interactions between a variety of diseases can be explained by the presence of overarching groups of co-occurring phenotypes. Shared susceptibility between such diseases can be derived from common genetic, biological, or environmental factors. Indeed, diseases with comparable characteristics can occur simultaneously or sequentially with similar pathogenesis in a subject (Skou *et al.* 2022). However, the best way to identify the contribution of genetic components to the etiology of such multimorbidities remains an open question. Due to the highly connected nature of diseases at the molecular level, it is necessary to concurrently examine not only phenotypes, but also the many genetic factors that could influence their pathological dynamics (Barabási *et al.* 2011). The field of network medicine offers an intuitive way of investigating the interactions between phenotypes (Sonawane *et al.* 2019). Both global and local connectivity across multiple phenotypes can be explored through graph-based modeling and network representation. In particular, the disease-disease network (DDN) represents diseases as nodes and connections between diseases, such as observed or quantified biological factors, as edges (Goh *et al.* 2007, Zhou *et al.* 2014). Earlier approaches for modeling shared disease mechanism relied on databases, synthesizing information from across literature. These databases were used to construct networks based on common disease-associated genes (Goh *et al.* 2007) or shared symptoms (Zhou *et al.* 2014). However, the increased availability of data in the modern era has enabled the development of less biased approaches to model multimorbidity relationships.

With the extensive growth of large-scale biomedical data, electronic health record (EHR)-linked biobanks have become a vital resource in the study of pleiotropy and the genetic architecture of complex traits. A phenome-wide association study (PheWAS) applied to an EHR-linked biobank can find hundreds of thousands of associations between phenotypes, such as diseases, clinical symptoms, or laboratory measurements, and genetic variants, such as common single-nucleotide polymorphisms (SNPs) (Denny *et al.* 2010). Furthermore, PheWASs are disease- and variant-agnostic, meaning that the identification of these potential instances of pleiotropy remains unbiased (Pendergrass *et al.* 2013, Hall *et al.* 2014). The summary statistics from a PheWAS can be used to create corresponding shared-SNP DDNs (ssDDNs), where edges represent sets of associated SNPs that pass a desired threshold of significance and are shared between the two phenotypes (Verma *et al.* 2019, Sriram *et al.* 2021, 2022). By analyzing a ssDDN, a researcher or clinician can evaluate how diseases are linked to one another, with immediate insight into potential shared genetic architecture through the identification of putative pleiotropic SNPs at specific genomic locations. ssDDNs built from UK Biobank (UKBB) PheWASs have accurately modeled known multimorbidities (Sriram *et al.* 2022), computed improved scores for disease complications through graph-based machine learning (Sriram *et al.* 2021), and generated outperforming individual genetic risk scores (Nam *et al.* 2022), using disease-SNP associations alone.

EHR-linked biobanks often report quantitative lab results of blood- and urine-based biochemical markers. Many of these traits have a strong genetic basis, and they can be used as intermediate phenotypes in the analysis of complex diseases, offering additional information in the investigation of disease connections (Wong *et al.* 2011, Kanai *et al.* 2018, Sinnott-Armstrong *et al.* 2021, Julkunen *et al.* 2023, Nag *et al.* 2023). Given the polygenic predictive power of such continuous endophenotypes, integrating them into studies of non-mendelian disorders allows for improved interpretability at the molecular level, beyond what genetic pleiotropy can uncover (Smith *et al.* 2022). Several individual laboratory measurements have been shown to be clinical predictors of cardiovascular disease, and evidence is accumulating for quantitative biomarker links with many other types of common diseases (Buergel *et al.* 2022). For example, Veturi *et al.* recently showed substantial pleiotropy between plasma lipids and diseases across many organ systems (Veturi *et al.* 2021). This is supported by over a decade of research from the Global Lipids Genetics Consortium, which has found that heritable lipid levels, such as lipoprotein cholesterols, triglycerides, and total cholesterol, are not only genetically related to complex diseases through shared loci, but are modifiable risk factors of those diseases (Teslovich *et al.* 2010, Willer *et al.* 2013, Liu *et al.* 2017).

Based upon the additional insight that may be derived from such intermediate phenotypes, we propose a novel augmented version of the ssDDN, denoted as ssDDN+. Additional genetic associations between diseases are incorporated into the original ssDDN based upon shared genetic correlation with clinical laboratory measurements. We hypothesize that a ssDDN+ can represent inherited factors contributing to cross-phenotype associations and provide insight into the role of endophenotypes in these disease interactions. In this study, we constructed a ssDDN+ using PheWAS summary statistics from the UKBB, revealing hundreds of genetic correlations between diseases and quantitative traits. We show that our augmented network uncovers genetic associations across different disease categories, connects relevant cardiometabolic diseases, and identifies specific biomarkers that are associated with the genetic architecture of multiple diseases. Comparing our ssDDN+ to its corresponding ssDDN demonstrates the complementary information that is revealed in this new network topology, highlighting the influence of quantitative traits within the diseasome (Goh *et al.* 2007).

## 2 Materials and methods

### 2.1 Data

PheWAS summary data from the UKBB were used to investigate the genetic relations among diseases (www.leelabsg.org/resources). To derive genetic associations for binary diseases, a PheWAS was run for 400 000 British individuals of European ancestry with 1403 phecode-labeled phenotypes using SAIGE (Zhou *et al.* 2018), controlling for sex, age, genetic relatedness, and the first four principal components. Imputation using the Haplotype Reference Consortium panel yielded 28 million imputed SNPs, with all genomic positions on GRCh37 (Gagliano Taliun *et al.* 2020). To improve interpretability and relevance of diseases under consideration, we removed phenotypes if they had a case count <1000 cases, had a phecode encoding specific to the hundredths digit, or belonged to phecode categories of ‘symptoms’ or ‘injuries & poisonings.’ The widely used 1000-case count threshold was established based on power to detect genetic associations (Zhang *et al.* 2019, Nam *et al.* 2023). The hierarchical structure of phecode classification defines diseases such that the closer the values are, the more similar diseases are physiologically. In this way, phecodes with the same hundreds digit are diseases part of the same organ system, e.g. carditis (420) and congestive heart failure (428), while diseases of the same integer are subtypes of the same diseases, e.g. type 1 diabetes (250.1) and type 2 diabetes (250.2). Phecodes specific to the hundredths digit represent hyper-specific diagnoses that are not different enough to capture unique genetic associations, and thus would add noise to the analysis of disease connections. Additional manual curation was applied to remove hierarchically related diseases with similar case counts that would have represented correlated signals, resulting in a final dataset of 318 binary diseases for the network.

To derive genetic associations for continuous endophenotypes, a PheWAS was run for 361 194 British individuals of European ancestry with 31 rank-normalized quantitative biomarker measurements (Supplementary Table S1). This PheWAS was performed for 13.7 million QC-passing SNPs using Hail 0.2 (Hail Team), corrected for sex, age, and the first 20 principal components (nealelab.github.io/UKBB_ldsc/downloads.html). Between the two PheWASs, for alleles to remain consistent across the full set of diseases and biomarkers, variants were restricted to a unified list of HapMap3 SNPs. Due to the complicated LD structure in the major histocompatibility complex, SNPs in that region were also removed (Finucane *et al.* 2015, Kanai *et al.* 2018). As a result, roughly 1.2 million SNPs remained for the identification of associations between diseases and laboratory measurements (Altshuler *et al.* 2010).

### 2.2 Disease–endophenotype correlations

The shared-SNP approach of identifying genetic associations between traits is a reasonable assumption for binary traits given the shared components hypothesis (Barabási *et al.* 2011). However, in the case of evaluating genetic associations between binary traits and continuous traits, such a method may fail to appropriately capture the magnitude of associations with the quantitative marker. Linkage disequilibrium score regression (LDSC) (Bulik-Sullivan *et al.* 2015b) offers an effective method of calculating genetic correlations between pairs of phenotypes through the analysis of PheWAS summary-level data (Bulik-Sullivan *et al.* 2015a). This process considers all common SNPs in a genome regardless of significance, accounting for SNP weight when determining associations between traits (Kanai *et al.* 2018, van Rheenen *et al.* 2019). LDSC importantly models both positive and negative relationships, while providing computational efficiency to implement across thousands of pairs phenotypes without, the need for individual level data. Applying LDSC to the summary statistics described above, we generated bivariate genetic correlation values (*r_g_*) between each binary disease and each quantitative endophenotype. Filtration to consider only genetic correlations for heritable phenotypes produced 9566 disease-endophenotype *r_g_* estimates. Of these correlations, 322 were found to be significant with a false discovery rate (FDR) < 0.05 (Benjamini and Hochberg 1995, Kanai *et al.* 2018, Kim *et al.* 2021).

### 2.3 Construction of ssDDN and ssDDN+

**Figure 1.**
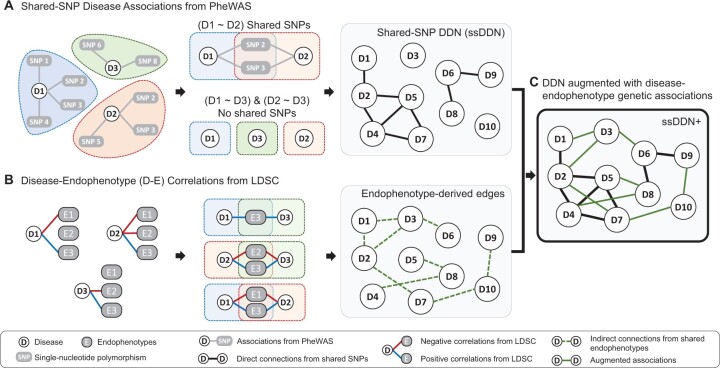
Overview of network construction. An overview of the process of developing the ssDDN+. (A) Diseases sharing genome-wide significant SNPs uncovered via a PheWAS are used to construct a shared-SNP DDN, where edges represent shared associations with variants between a pair of diseases. (B) Genetic correlation is determined between all diseases and quantitative endophenotypes, and if diseases are both genetically correlated (dashed line) with the same endophenotype then edges between those diseases are added to the ssDDN. (C) Design of the combined networks as a ssDDN+

Curated PheWAS summary data for the 318 binary diseases were used to generate the baseline ssDDN. The augmented version of the ssDDN, the ssDDN+, was constructed by incorporating the same PheWAS summary data with genetic correlations between the 318 diseases and the 31 aforementioned rank-normalized quantitative biomarker measurements (Fig. 1). These 31 endophenotypes, including lab measurements like Albumin, HDL-C, and Vitamin D, were all included in the ssDDN+ due to their inherent correlation with various diseases (Sinnott-Armstrong *et al.* 2021). Our selection was limited to this set of 31 traits due to data availability constraints. The methodology described by Verma *et al.* (2019) was applied to create the ssDDN. A minor allele frequency threshold was set at > 0.05 to ensure power to detect genetic signal. An edge in the set $E={\left\{e_{ij}\right\}}^{\left|V\right|\times |V|}$ was established between each pair of binary phenotypes $v_{i}$ and $v_{j}$ if the two diseases shared associations with at least one common SNP at a genome-wide significance threshold of $5\times 10^{-8}$ (Sriram *et al.* 2021, 2022, Nam *et al.* 2022, 2023). $e_{ij}$ represents the presence or absence of a connection, meaning that $e_{ij}=1$ if $v_{i}$ and $v_{j}$ had any common shared SNPs and $e_{ij}=0$ otherwise. These edges can be thought of as direct links between phenotypes in the ssDDN. The final ssDDN is an undirected, unweighted graph.

The corresponding ssDDN+ can be represented as graph $\tilde{G}=(V,\tilde{E})$, where node set V represents the set of binary phenotypes and edge set $\tilde{E}$ represents all connections between phenotypes. $\tilde{E}$ can be decomposed into direct connections (E) obtained from the ssDDN and indirect connections ($E^{+}$) estimated from significant genetic correlations derived from LDSC. We constructed a genetic correlation matrix $R={\left\{r_{ij}\in R\right\}}^{\left|V\right|\times |T|}$ where T represents the set of all quantitative traits. The correlation matrix R was transformed into an association matrix $\hat{R}$, such that ${\hat{r}}_{ik}=1$ if the genetic correlation $r_{ik}$ between phenotype $v_{i}$ and quantitative trait $t_{k}$ passed statistical significance. Then, the indirect connection ($e_{ij}^{+}$) was established by determining whether phenotypes $v_{i}$ and $v_{j}$ shared a genetic association with the same trait $t_{k}$ with $e_{ij}^{+}=\mathrm{sgn}({\hat{r}}_{ik}\cdot \;{\hat{r}}_{jk})$, where sgn(⋅) is the signum function. If a common genetically correlated quantitative trait was identified between the two phenotypes, then an indirect edge was included in $E^{+}$. Since an unweighted edge is incorporated into the ssDDN+ regardless of the number of associated biomarkers, correlations between the quantitative traits did not affect the generated ssDDN+. The final graph, with edge set $\tilde{E}$ comprising of the union of E and $E^{+}$, corresponds to the complete undirected, unweighted ssDDN+. To generate, visualize, and analyze both graphs, we made use of Gephi 0.90 (Bastian *et al.* 2009) and sigma.js (Sigma.js), open-source network visualization software packages, as well as NETMAGE (Sriram *et al.* 2022), a web-based tool that allows users to upload PheWAS summary statistics and generate corresponding interactive DDNs. Further analysis and visualization of DDN network statistics were performed using R 4.1.3 (R Core Team 2022).

### 2.4 Disease categories

The 318 phecode-encoded binary phenotypes were organized into 15 unequally sized categories (Supplementary Table S2) (Wu *et al.* 2019). These standardized categories representing organ systems and other relationships were defined by the established phecode chapters, derived from the original ICD-9 chapters (Wei *et al.* 2017, Bastarache 2021). Category-specific analyses allowed us to assess how the network structure of the ssDDN+ can provide insight into connections between biologically similar diseases that affect the same organ systems. We considered phenotypes in the groups ‘endocrine/metabolic’ and ‘circulatory system’ as cardiometabolic diseases.

## 3 Results

### 3.1 Additional edges in the ssDDN+

**Figure 2.**
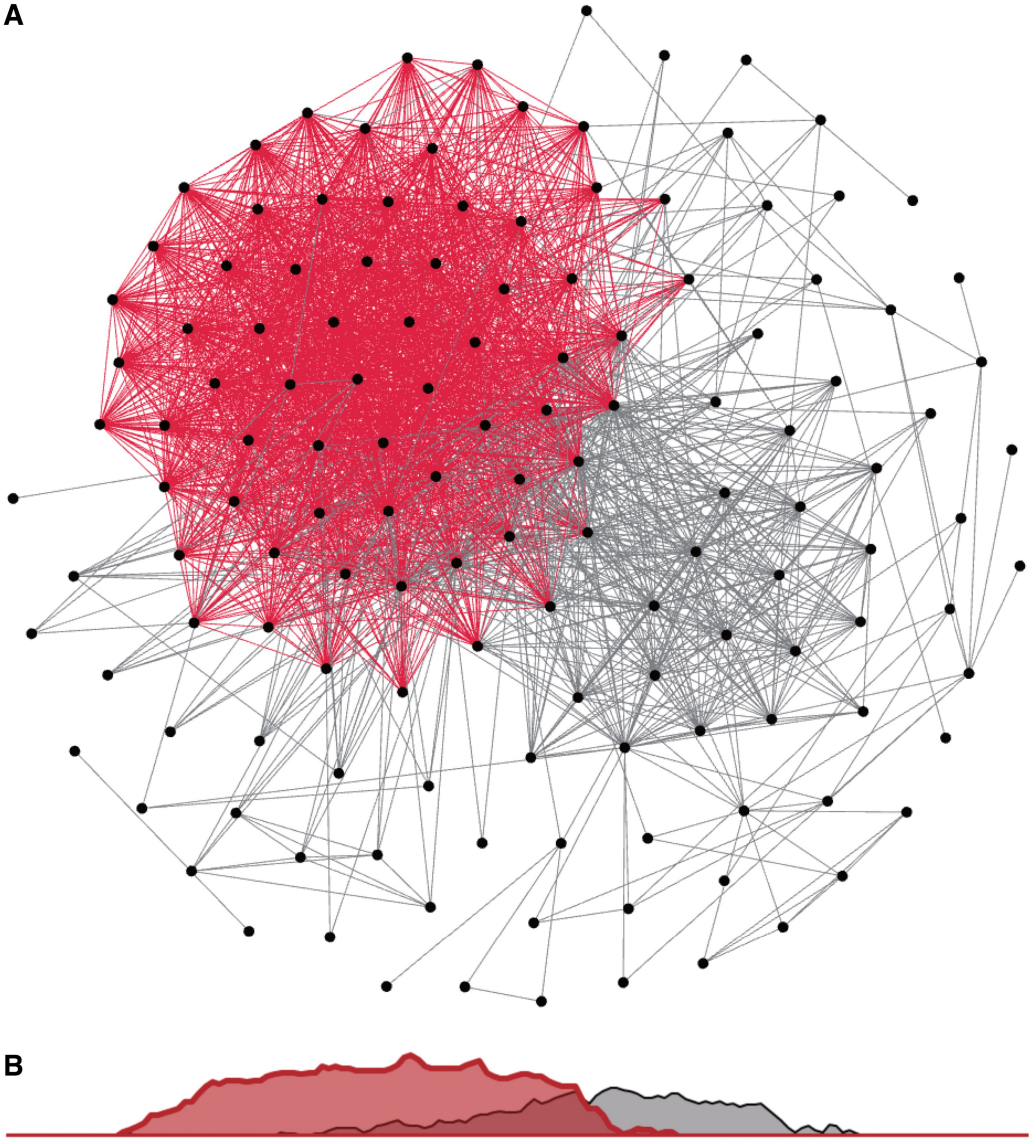
Endophenotype augmented DDN. (A) A depiction of the full ssDDN+ based upon PheWAS summary statistics of binary diseases and continuous biomarker measurements from the UKBB. Gray edges represent direct shared-SNP edges, and red edges represent indirect biomarker genetic correlation edges. (B) A density plot projection of direct and indirect edge distributions in a single dimension. Direct and indirect edges identify different sets of genetic associations between diseases

Using the 322 genetic correlations between binary diseases and continuous measurements (Supplementary Figs S1 and S2), we constructed a corresponding ssDDN+ from our UKBB ssDDN (Fig. 2). 1561 new cross-phenotype genetic associations were identified compared with the original ssDDN, increasing the network’s total edge count by 242% (Supplementary Table S3). Out of the 31 continuous measurements under consideration, 21 of them were genetically correlated with at least one disease. The ssDDN and ssDDN+ exhibited similar clustering behavior to one another (Supplementary Table S4). However, including indirect edges increased the connected node count from 114 to 138, meaning that 24 diseases gained connections to others because of associations derived from laboratory measurements. 116 indirect edges represented the same cross-phenotype associations as pre-existing direct edges, suggesting that highly significant SNPs associated with disease associations may be involved in the same pathways as the biomarkers that connect them. Indirect edges that contributed new information in the ssDDN+ can be explored online through our Human-Disease Phenotype Map browser at hdpm.biomedinfolab.com/ddn/biomarkerDDN. Additional network statistics for each DDN can be found in Supplementary Table S4.

### 3.2 Highly connected diseases and hub nodes

**Figure 3.**
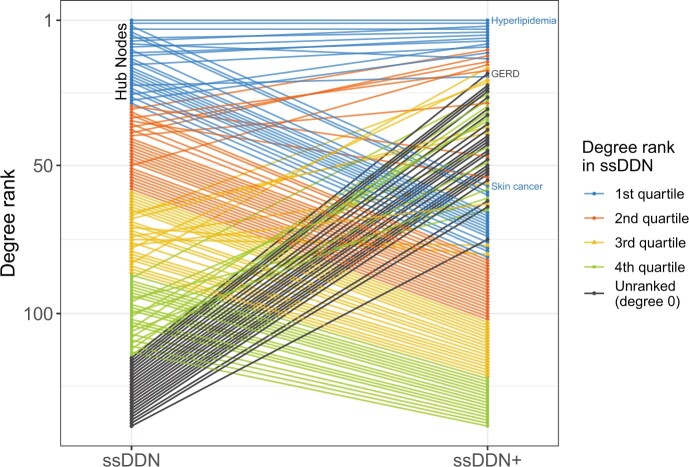
Change of node degree rank from ssDDN to ssDDN+. A slope graph of degree rankings for diseases in the ssDDN and ssDDN+. The degree of a node in a graph represents the number of other nodes to which it is connected. Within each network, degrees were computed for each node, and then diseases were ranked with respect to one another according to degree value. A rank of 1 represents the most connected disease. For both DDNs, hyperlipidemia (phecode 272.1) has the highest node rank. Ranks in the figure are colored by quartile within the ssDDN, with black representing nodes that became connected in the ssDDN+ after not having any connections in the original ssDDN. Some newly connected nodes (e.g. GERD) are hub nodes in the ssDDN+, while some highly connected nodes (e.g. skin cancer) became relatively less connected

Within each DDN, a node’s degree, the number of other nodes to which it is connected, represents how genetically associated the corresponding disease is to other diseases. Hub nodes, nodes with the highest centrality in the graph, represent the most highly connected diseases. When we transition from the ssDDN to the ssDDN+, the relative degree of many diseases changes substantially. Figure 3 demonstrates how the degree rank of diseases changes by supplementing the ssDDN with indirect edges and highlights known biology and genetic susceptibility for certain diseases. For instance, hyperlipidemia, a disease whose signal in our data is mostly represented by patients with hypercholesterolemia, has known mendelian effects from genes including *LDLR*, *APOB*, and *PCSK9* (Vrablik *et al.* 2020). Correspondingly, we see hyperlipidemia has the top degree rank in the ssDDN. Furthermore, hyperlipidemia also exhibits known associations with a variety of lipidomic biomarkers (Rai and Bhatnagar 2017), justifying its role as the disease with the highest degree in the ssDDN+.

Many newly connected diseases also gain a high degree rank compared to other diseases after the inclusion of endophenotypes to the ssDDN. For instance, gastroesophageal reflux disease (GERD) has a known heritability estimate of roughly 31% based upon twin and family studies, with known risk genes including *FOXF1*, *MHC*, and *CCND1* (Argyrou *et al.* 2018). However, the original ssDDN fails to capture any sort of genetic signal for GERD, meaning that the disease remains unconnected to other nodes. This failure to identify cross-phenotype associations with GERD in the original ssDDN is likely due to a combination of stringent significance thresholds for disease-variant association as well as external factors outside of genetics mitigating the associations that would otherwise be apparent in the input PheWAS data. External risk factors for GERD include elements such as age, body mass index, smoking status, eating and sleeping habits, and other sociodemographic variables (Clarrett and Hachem 2018). Given known evidence that predictive biomarkers, such as C-peptide and TNF-alpha (Haider *et al.* 2018), exist for GERD, this disease becomes a perfect candidate for the identification of additional information in the ssDDN+. Indeed, based upon the inclusion of endophenotypes, GERD gains one of the highest degree ranks in the ssDDN+.

Finally, some diseases that are originally hub nodes in the ssDDN become comparatively less influential in the ssDDN+. For instance, skin cancer is a hub node in the ssDDN and is known to have common genetic associations with a variety of other neoplasms (Frank *et al.* 2017). However, skin cancer prognosis is not improved through the analysis of biomarkers (Deacon *et al.* 2021). This behavior is accurately reflected in both networks in our study. Within the ssDDN, skin cancer has a prominent position with respect to other diseases in the network. From the measures that we incorporate into our ssDDN+, no additional edges are included for skin cancer. Thus, as expected, this specific ssDDN+ provides no further information about cross-phenotype associations with skin cancer as compared to its corresponding ssDDN.

### 3.3 Differential contribution of endophenotypes by phenotype category

**Figure 4.**
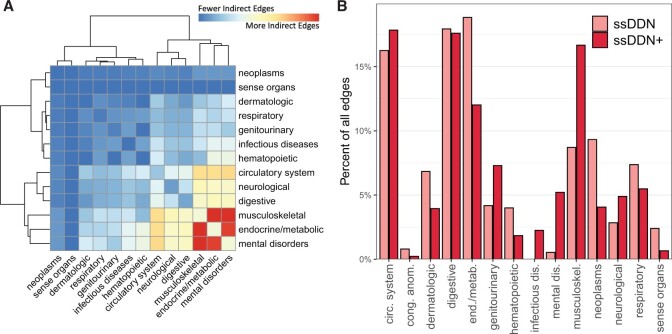
Category distribution of indirect edges. (A) A heatmap of the disease categories connected by indirect edges in the ssDDN+, normalized by the number of nodes in each category. Hot colors represent category pairs with more indirect edges, and cold colors represent category pairs with fewer indirect edges. (B) A paired bar chart depicting the percentage of edges connecting at least one node in each disease category, colored by type of DDN. Some categories gain a disproportionately large number of edges from ssDDN to ssDDN+, while others gain only few edges

Although the addition of new edges in the ssDDN+ changes the topology of the network, this change is not evenly distributed across organ systems and disease types. Figure 4a depicts specific pairs of phenotype groupings that become increasingly connected to one another by these new edges. In particular, a high concentration of new edges between the musculoskeletal and endocrine/metabolic disease categories is observed. This behavior is corroborated by prior research indicating associations between musculoskeletal degradation and the onset of metabolic disorders (Collins *et al.* 2018). On the other hand, disease categories such as neoplasms and sense organs continue to remain relatively disconnected to other groupings, confirming conclusions drawn regarding cross-phenotype associations across disease categories in previous studies (Goh *et al.* 2007, Verma *et al.* 2019). These differences across disease categories are due in part to the types of diseases that are genetically associated with the clinical measurements for which we had data to use. Indeed, we observe noticeable changes in the proportion of edges connected to diseases depending on category in the ssDDN+ (Fig. 4b). The most notable difference is the relative doubling of links connected to the phenotypes in the musculoskeletal system. Additionally, the proportion of edges that connect diseases from different groups increases from 75% to 85%, suggesting that endophenotypes may be useful in identifying additional genetic associations between diseases of different categories.

### 3.4 Cardiometabolic disease associations and influence of HDL-C

**Figure 5.**
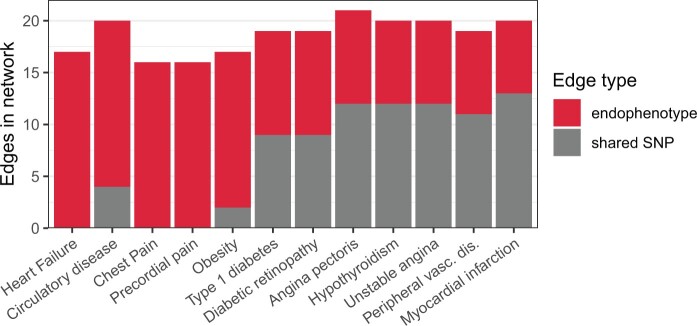
Cardiometabolic network edge types. A stacked bar chart depicting the types of links connected to the 12 diseases that gained the most edges going from the cardiometabolic ssDDN to the cardiometabolic ssDDN+. Gray on the bottom represents direct shared-SNP edges, and red the on top represents indirect endophenotype-correlated edges. Some diseases with known genetic drivers become connected to other phenotypes only as a result of indirect edges. For instance, clinical symptoms including heart failure, chest pain, and precordial pain, can only be connected to other chronic diseases after augmenting the ssDDN with endophenotypes

Previous research has highlighted a variety of potential genetic contributors to comorbidities among cardiometabolic diseases (Faner *et al.* 2014, Locke *et al.* 2015, Cruz-Ávila *et al.* 2020), and an initial analysis of the ssDDN+ seems to confirm the influence of the endocrine/metabolic disease category. To further investigate such connections, we focus in on a subnetwork of our ssDDN+, where we consider only cardiometabolic phenotypes. The inclusion of 144 endophenotype genetic correlations increases the edge count from 116 to 200 when transitioning from the cardiometabolic ssDDN to its ssDDN+ (Supplementary Fig. S3). Multiple diseases of great interest, including heart failure, obesity, and type 1 diabetes, also become much more genetically connected, suggesting that in many instances, important disease connections may be missed in the ssDDN (Fig. 5).

**Figure 6.**
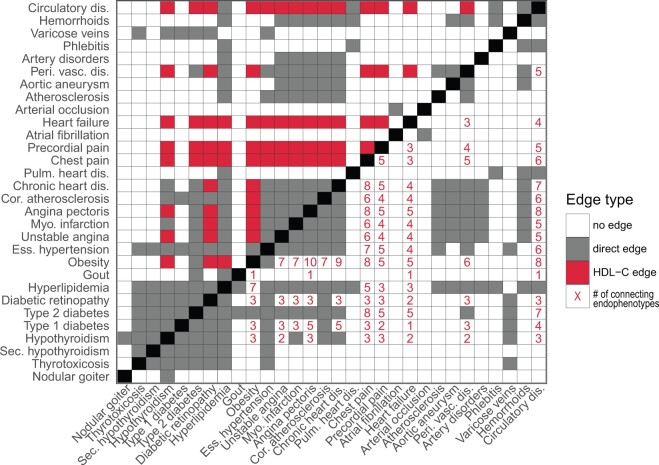
Contribution of edges by individual endophenotypes. An adjacency matrix presenting the contribution of HDL-C to edges in the cardiometabolic ssDDN+, as well as the influence of different clinical traits on connections among diseases. Gray squares represent associations identified between diseases through the shared SNP approach, while white squares represent a lack of connection between phenotypes. In the upper triangular adjacency matrix, red squares represent disease associations generated as a result of HDL-C. In the lower triangular matrix, red numbers represent the number of biomarker correlations shared between diseases in the ssDDN+

The clinical traits used to build the ssDDN+ are involved in many different pathways, and thus we find certain biomarkers reveal many more edges than others. For instance, high-density lipoprotein cholesterol (HDL-C) contributes 996 new edges in the full ssDDN+ and 70 new edges in the cardiometabolic ssDDN+, while other biomarkers such as phosphates add no new edges (Supplementary Tables S5 and S6). This result highlights how clinical biomarkers may provide different levels of information from shared SNP links, and how phenotypes such as HDL-C may offer improved predictive power in identifying disease comorbidities. Focusing in on the cardiometabolic-specific ssDDN+, we can visualize how HDL-C adds considerable edges to the network. (Fig. 6). For instance, the inclusion of genetic correlation through HDL-C as edges connects hypothyroidism and angina pectoris, diseases known to be associated with HDL-C and with one another (Ellyin *et al.* 1992, Tselepis *et al.* 1996).

## 4 Discussion

In this study, we generated and analyzed a DDN of genetic associations between binary phenotypes using significant SNPs from PheWAS summary statistics and genetic correlations with clinical laboratory measurements. Our network complements others by uncovering cross-phenotype links through genetic correlations between diseases and biomarkers, creating a denser model of the phenome. We highlighted disease classes as well as specific diseases with known genetic risk which benefit from this type of representation. Further analysis of the cardiometabolic DDN determined that this method enhances the clinical understanding of disease connections.

Studies of missing heritability throughout the past decade have made it apparent that considering only highly significant GWAS SNPs will often fail to capture the entire genetic architecture of complex diseases (Zuk *et al.* 2012, Crouch and Bodmer 2020). It is additionally important to functionally assess genetic effects—understanding the association between diseases and the disruption of molecular pathways through mutations can bring us closer to fully comprehending how diseases manifest as comorbidities and complications (Girirajan 2017). Both points highlight the utility of incorporating disease-associated biomarkers into the formation of human disease networks. Furthermore, PheWASs based on logistic regression binarize complex diseases that may have a range in their physical manifestation, making the use of endophenotypes even more pertinent.

In our analysis, the endophenotypes we incorporated contribute a non-random distribution of edges to specific diseases categories—musculoskeletal diseases gain more connections, while neoplasms gain much fewer. This difference is driven in part by which types of diseases have significant correlation with the biomarkers under consideration, and how for some phenotypes, the analysis of biological molecules is more useful when assessing genetic contributors. The differential augmentation across diseases provides evidence of the importance of including quantitative laboratory measurements. When looking specifically at cardiometabolic phenotypes, new edges are added from associations with biomarkers to phenotypes that have been determined to have strong polygenic causes, including heart failure (Czepluch *et al.* 2018), obesity (Loos and Yeo 2022), and diabetic retinopathy (Cho and Sobrin 2014).

The fundamental value this ssDDN+ adds is a novel way to model the diseasome. There has been substantial work exploring cross-phenotype associations to identify shared architecture among human diseases (Canela-Xandri *et al.* 2018, Gagliano Taliun *et al.* 2020, Wang *et al.* 2021). But by harnessing the value of intermediate phenotypes, we can represent an increased number of genetic associations present in disease connections. For example, this ssDDN+ links Heart Failure (phecode 428.2), a disease with no connections in the ssDDN, to 54 other diseases. By integrating genetic correlations with endophenotypes into the ssDDN, we pick up additional signal that make the investigation of this and other phenotypes’ connections possible. Our network has multiple potential future applications, including drug design with network pharmacology, finding genetic targets for future therapeutics, and the advancement of personalized medicine and disease risk prediction (Chandran *et al.* 2017). In particular, by modeling the nonlinear and interactive genetic relationships between diseases and endophenotypes, we built a framework that can be used as input for genetic risk prediction (Nam *et al.* 2022), that aligns closer with the biology of the disease systems. The ssDDN+ also provides enhanced interpretability of such risk scores, through the combination of representing multimorbidities with diseases and the relevant blood markers.

There are a few limitations to consider in our study. Though the binary diseases and continuous laboratory measurements both come from the UKBB, the summary statistics for each category of traits were generated by different groups with different processing conditions, yielding slightly different numbers of individuals in each case. Within the binary disease PheWAS however, each GWAS uses a slightly different number of samples due to phenotype-specific exclusion criteria. Therefore, these relatively small differences in samples across PheWASs should not undermine our results. The two PheWASs also use slightly different criteria to define their SNPs, with one having around 13.7 million SNPs tested compared to roughly 28 million SNPs in the other. Since we harmonized the SNPs down to a count of 1.2 million variants with precomputed LD scores, this distinction does not impact our analysis. Additionally, we used very strict significance thresholds, both for finding shared SNPs between diseases and for determining legitimately genetically correlated biomarker-phenotype pairs. Although this stringency may result in missing some genetic associations between diseases, it allows us to be confident in the connections we do observe in the ssDDN. Another consideration of this study is that the genotypes analyzed in our networks are based on GRCh37—in the future, with more available data, our methods can be applied to datasets based on GRCh38, in keeping with other recent work in the field of genetics. We also note that our DDNs represent data only for the UKBB population, meaning that conclusions drawn from our analysis can only be interpreted from a British European perspective. In the future, when additional large-scale PheWAS data become available from biobanks such as the Million Veterans Program or the All of Us biobank, validation analyses can also be performed to compare the structure of our generated UKBB DDNs. Further validation can be evaluated by comparing the network structure with connections derived from other biological information, like shared interacting proteins, or shared pathways (Dong *et al.* 2021). Furthermore, as more biochemical markers become available in biobanks, a more comprehensive PheWAS of relevant endophenotypes can be employed, narrowing down the precise molecules related to disease multimorbidities. Finally, despite the fact that the phecode system of disease classification is more aligned with definitions of biomedical research than comparable disease encoding systems such as ICD-9 or ICD-10 (Wei *et al.* 2017), we appreciate that it is still imperfect at capturing true occurrences of phenotypes in patients. As a result, any conclusions drawn from our analysis need to bear this potential inaccuracy in mind.

In conclusion, we built an augmented DDN that integrates genetic correlations with endophenotype measurements to represent additional cross-phenotype associations. Further steps in our analysis involve considering additional clinical traits depending on data availability, as well as additional population cohorts, as we may find even more endophenotype associations and thus more network edges(Sun *et al.* 2022). We also hope to compare ssDDN+s to their corresponding ssDDNs given different significance thresholds for associations between diseases and SNPs. Future work should consider integrating mendelian randomization to identify the causality behind the correlative relationships that were uncovered. Additionally, full analysis of networks built from all levels of diseases risk (SNP-based, gene-based, symptom-based, molecular-based, pathway-based, microRNA-based, exposure-based, etc.) will be essential to integrate into studies and patient-prediction tasks (Jin *et al.* 2019), along with multilayer graphs that summarize complex biological architecture beyond individual edges. Overall, our method helps to navigate the study of complex diseases and enables further network-based analysis involving pleiotropy, polygenicity, and heterogeneity. Our results can facilitate future network-based research of diseases, uncovering potential sources of missing heritability in multimorbidities and highlighting potential genetic targets for precision medicine investigations.

## Supplementary Material

btae126_Supplementary_Data

## Data Availability

Summary statistics for binary diseases can be accessed at https://www.leelabsg.org/resources and for continuous laboratory measurements at https://nealelab.github.io/UKBB_ldsc/downloads.html. Additionally, new endophenotype disease connections can be explored on our web visualization tool at https://hdpm.biomedinfolab.com/ddn/biomarkerDDN.
