# Supplementary material for: Uncovering genetic associations in the human diseasome using an endophenotype-augmented disease network
Source: Bioinformatics. 2024 Mar 12;40(3):btae126. doi: 10.1093/bioinformatics/btae126 (PMC10963079; doi:10.1093/bioinformatics/btae126)
Supplement: btae126_Supplementary_Data [file btae126_supplementary_data.zip › bioinformatics_ssDDN+_supp_info.pdf]

# Supplemental information

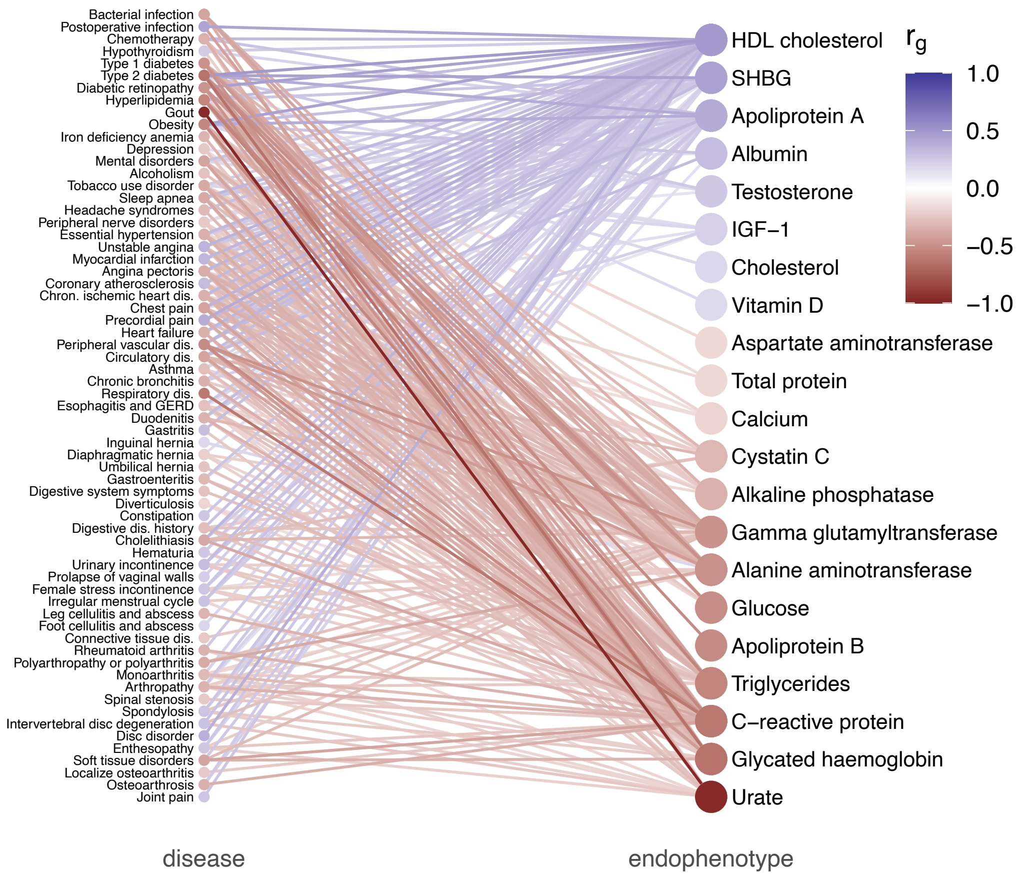


**Fig. S1: Bipartite graph of genetic correlations between diseases and endophenotypes**
A bipartite graph showing all 322 genetic correlations between binary disease phenotypes and quantitative measurements. Edges are colored according to correlation value.

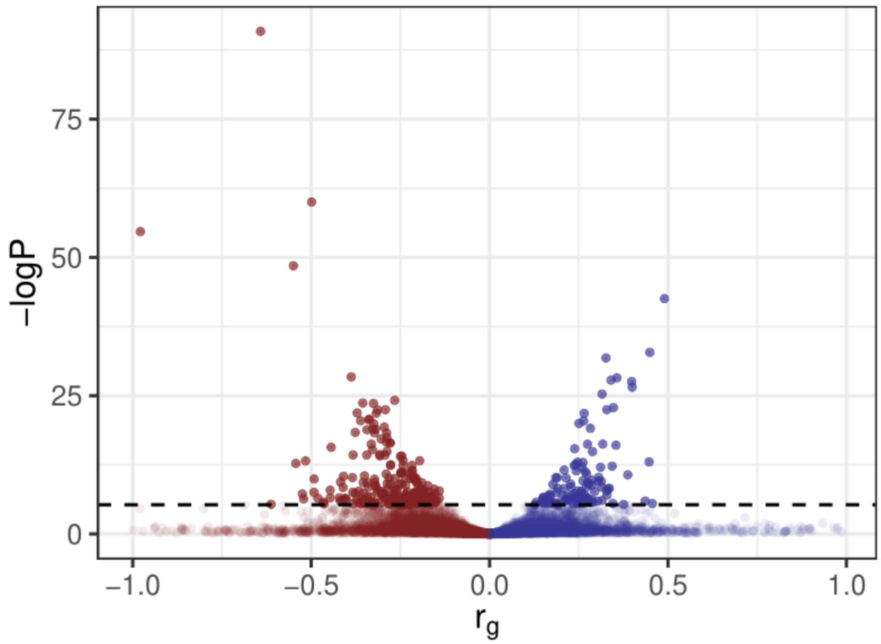


**Fig. S2: Distribution of genetic correlations between binary diseases and endophenotypes**

A volcano plot showing significance level against $r_g$ for all computed genetic correlations between heritable clinical measurements and disease phenotypes. The 322 significant genetic correlations are highlighted, along with the threshold of the correction factor (FDR < 0.05). There is an even distribution of positive and negative correlations.

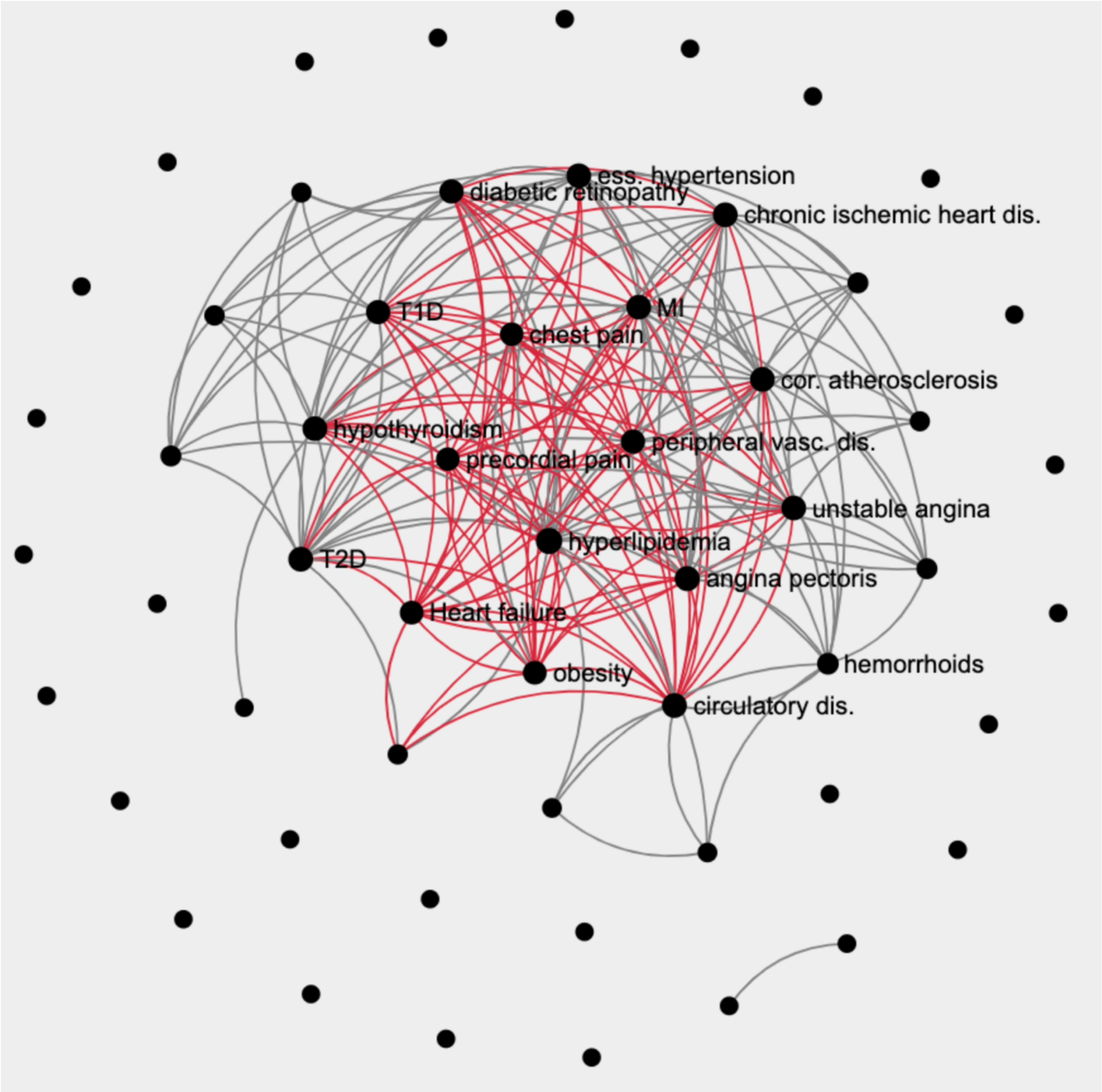


**Fig. S3: Cardiometabolic ssDDN+**

A subnetwork of the full ssDDN+ including only endocrine/metabolic and circulatory system diseases, colored by edge type: gray for direct and red for indirect. Phenotypes gaining the most edges through genetic correlation with quantitative endophenotypes are labeled in the network.
